# Impact of Single *versus* Double Clamp Technique on Blood Lactate Levels and Postoperative Complications in Coronary Artery Bypass Surgery

**DOI:** 10.21470/1678-9741-2020-0025

**Published:** 2022

**Authors:** Rifat Özmen, Muhammet Bozguney, Ali İhsan Tekin, Tamer Eroglu, Aydin Tuncay

**Affiliations:** 1 Department of Cardiovascular Surgery, Erciyes Universitesi Tip Fakultesi, Kayseri, Turkey.; 2 Department of Cardiovascular Surgery, Kayseri Egitim ve Arastirma Hastanesi, Kayseri, Turkey.; 3 Department of Cardiovascular Surgery, Ömer Halisdemir University, Nigde, Turkey.

**Keywords:** Cardiopulmonary Bypass, Retrospective Studies, Incidence, Lactic Acid, Mammary Arteries, Constriction, Coronary Artery Bypass, Stroke, Postoperative Complications

## Abstract

**Introduction:**

Cardiopulmonary bypass (CPB) is associated with hyperlactatemia, which leads to adverse clinical outcomes. No study has examined the effect of different clamping techniques on postoperative hyperlactatemia (PHL). Thus, we aimed to evaluate the impact of two different techniques on PHL and the clinical outcomes in patients undergoing isolated coronary artery bypass surgery.

**Methods:**

This retrospective study included 100 patients who underwent isolated CPB either with single clamp technique (SCT, n=47) or double clamp technique (DCT, n=53). Demographic and preoperative laboratory data, as well as operative features and arterial blood lactate levels at the onset and at the end of CPB, were collected from patient charts.

**Results:**

Blood lactate levels collected at the end of CPB did not differ significantly between groups whereas intraoperative lactate increased significantly in both groups (*P*<0.005). PHL developed in 16 patients (32%). There was no meaningful difference in SCT and DCT in this regard. Left internal mammary artery was used more frequently in the DCT group than in the SCT group. While the cross-clamp time was significantly longer in the SCT group, there was no difference regarding CPB time. Among postoperative complications, only the incidence of stroke was significantly higher in the DCT group than in the SCT group (10.6% *vs*. 0%, *P*=0.020). CPB time, cross-clamp time and numbers of proximal saphenous graft and distal anastomosis showed a significant positive correlation with the postoperative lactate level. In the regression analysis, CPB time emerged as the only independent predictor of PHL (OR 1.04, CI 95% 1.01-1.07, *P*=0.011).

**Conclusion:**

There was no difference in postoperative blood lactate levels between SCT and DCT groups.

**Table:** 

Abbreviations, acronyms & symbols				
**AF**	**= Atrial fibrillation**		**LIMA**	**= Left internal mammary artery**
**CABG**	**= Coronary artery bypass grafting**		**MDRD**	**= Modification of Diet in Renal Disease**
**CPB**	**= Cardiopulmonary bypass**		**OR**	**= Odds ratio**
**DCT**	**= Double clamp technique**		**PHL**	**= Postoperative hyperlactatemia**
**ECG**	**= Electrocardiogram**		**SCT**	**= Single clamp technique**
**ICU**	**= Intensive care unit**		**SVG**	**= Saphenous vein graft**
**LAD**	**= Left anterior descending**		**XCL**	**= Cross-clamp**
**LDL**	**= Low-density lipoprotein**			

## INTRODUCTION

Lactate is the end product of glucose metabolism. Lactic acid is the primary biologic form at physiological pH. The blood lactate level under normal physiological conditions is stable and balanced. However, several pathophysiological conditions impair this balance and, consequently, cause hyperlactatemia and acidosis^[1]^.

Open-heart surgery, particularly when carried out with cardiopulmonary bypass (CPB), is associated with the development of hyperlactatemia^[1]^. The hyperlactatemia seen in patients undergoing CPB is multifactorial. More importantly, several studies have clearly shown that hyperlactatemia is associated with poorer postoperative clinical outcomes on different types of heart surgery^[2]^.

Several independent risk factors are associated with the development of hyperlactatemia during CPB. These include longer CPB and cross-clamp times, need for inotropic support, need for blood transfusion, hypoxia, and low cardiac output^[3,4]^.

Generally, two aortic clamping techniques are used while constructing proximal and distal anastomoses during CPB. Only one aortic cross-clamp (XCL) is used in the single clamp technique (SCT), whereas one extra clamp in addition to the aortic cross-clamp, called partial side-biting clamp, is used in the double clamp technique (DCT)^[5]^. Several studies have compared these two techniques in terms of postoperative complications. Among them, most found that SCT is more favorable with respect to postoperative stroke^[5-7]^. These studies did not mention any meaningful differences in the rates of other postoperative complications.

To the best of our knowledge, although there are several studies on these clamping techniques, none of them have mentioned or emphasized hyperlactatemia in patients undergoing CPB. Since aortic clamping time may affect the development of hyperlactatemia, we hypothesized that the use of these two different clamping methods might have an impact on the development of hyperlactatemia. Thus, we aimed to evaluate the effects of SCT and DCT on blood lactate levels with other postoperative complications retrospectively in patients who underwent isolated coronary artery bypass surgery. Additionally, when performing proximal anastomoses, we aimed to investigate whether two different clamping techniques will have any effect on blood lactate level over CPB time.

## METHODS

This was a retrospective file review aimed at investigating the effect of single or double clamp proximal anastomosis technique on blood lactate levels and postoperative complications in patients who underwent isolated coronary artery bypass grafting (CABG) surgery. The Ethics Committee of the Erciyes University approved the study protocol (2019\371).

### Patients

We retrospectively screened adult patients aged between 35 and 90 years old who underwent CABG via open-heart surgery at our institution. All patients were operated on by the same surgical team. All patients who underwent elective isolated CABG between July 2018 and February 2019 were included in the study. The exclusion criteria were concomitant valve repair or aortic surgery, emergency surgery, previous open-heart surgery of any kind, preoperative blood lactate level above normal, ascending aorta calcification detected on echocardiography or chest radiography in the preoperative period, serum troponin level above normal or percutaneous coronary intervention in the previous week and antiaggregant treatment, preoperative atrial fibrillation, left ventricular aneurysm and/or thrombus formation, in addition to patients operated on by a different surgical team, and off-pump cardiac surgery.

A total of 280 patients were scanned and 100 patients were included after applying the exclusion criteria. The surgical operation notes were collected for each patient and the whole study cohort was divided into two groups: Group 1 included patients whose proximal anastomoses were performed using a single clamp technique (SCT, aortic cross-clamp only) and Group 2 included patients whose proximal anastomoses were performed by double clamp technique (DCT, aortic cross-clamp + side-biting clamp). The choice of clamp technique for the creation of proximal anastomoses was determined at the operating surgeon's discretion. The selection of the clamping technique used for the formation of proximal anastomoses was determined from the surgical notes as it is a retrospective study.

### Data Collection

Patient charts were retrospectively evaluated and demographic features, including age, gender, comorbidities including chronic obstructive pulmonary disease, diabetes mellitus, hypertension, carotid stenosis, peripheral arterial disease and chronic kidney disease, were considered. In addition, preoperative laboratory data, including serum hemoglobin, white blood cell count, neutrophil count, platelet count, hemoglobin A1C, serum creatinine, blood urea nitrogen, low-density lipoprotein (LDL) cholesterol and albumin, were recorded for each case. The glomerular filtration rate was calculated using the Modification of Diet in Renal Disease (MDRD) Study equation for each patient. All patients underwent transthoracic echocardiography (Philips ClearVue 550) performed by a cardiologist. Arterial blood lactate levels were measured for five minutes before the cross-clamp and ten minutes after the end of CPB. As defined in the literature, hyperlactatemia was considered when blood lactate levels were> 3 mmol/l^[3,8]^. Surgical and anesthetic monitoring notes were assessed to extract data regarding to intraoperative features, such as the status of left internal mammary artery (LIMA) graft use, the number of proximal and distal anastomoses, cross-clamp and CPB times, intraoperative mean blood pressure and need for inotropic support, preoperative and postoperative glucose, bicarbonate and pH values.

Postoperative cerebrovascular accident (stroke) was defined as the development of a new focal neurological deficit (motor weakness, visual impairment, dysarthria/dysphasia etc.) that requires a neurologic evaluation by a neurologist and the presence of a commensurate lesion on imaging studies, such as brain computed tomography and magnetic resonance imaging. Patients who had atrial fibrillation (AF) documented with ECG irrespective of returning to sinus rhythm and who were administered amiodarone and/or low molecular weight heparin after the operation were recorded as having postoperative AF. Postoperative surgical site of infection was defined as any infection in the surgical area (in the sternotomy or graft harvesting areas) that required infectious diseases consultation and commencement of appropriate antibiotics based on positive culture results.

Study groups were compared in terms of intraoperative lactate increase, length of intensive care unit and hospital stay, amount of bleeding (discharge) during the first postoperative day, need for revision surgery, need for inotropic support more than three hours after the operation and rates of cerebrovascular accident, *de novo* AF, surgical site infection, and mortality. In addition to any potential effect of the clamping technique on postoperative lactate level, we also aimed to assess whether there was any association between postoperative lactate levels and any of the study parameters mentioned above.

### Surgical Procedures

A median sternotomy was performed under general anesthesia after right radial artery cannulation and right central jugular venous catheter placement in all patients. Saphenous vein graft (SVG) for all patients and LIMA graft for patients who did not have a contraindication were prepared. Standard aortic and dual-stage venous cannulation was performed. After initiation of CPB, all patients were cooled up to 31 °C. The XCL was placed, and ice water was used for topical hypothermia. Cold blood cardioplegic solution was infused with an average pressure of 80-120 mmHg via CPB. At 20-minute intervals, maintenance blood cardioplegic solution was infused.

Between these 20-minute intervals, distal anastomoses were performed first. For patients who received LIMA grafts, LIMA-LAD anastomosis was performed as the last anastomosis.

After all distal anastomoses were completed, a final cold blood cardioplegic solution was administered to patients who underwent proximal anastomosis with a cross-clamp. When proximal anastomoses were completed, patients were rewarmed to 34.5 °C and a warm blood solution was infused via CPB under a mean pressure of 80-120 mmHg. Then, the cross-clamp was removed. Afterwards, the flow rate was reduced and patients who reached appropriate blood pressure and arterial blood gas analysis were weaned from CPB. Patients with insufficient cardiac contractions were not weaned from the machine, and patients whose blood pressures did not increase enough or decrease while the flow rate decreased received inotropic support.

Patients whose proximal anastomoses were performed under side-biting clamp (double clamp group) were rewarmed up to 33.5 °C, and a warm solution was infused via CPB under a mean pressure of 80-120 mmHg. After that, the cross-clamp was removed. Then, a side-biting clamp was placed. Proximal anastomoses were performed. Following the completion of proximal anastomoses, patients were weaned from CPB when appropriate blood pressure, body temperature, and blood gas values were reached. As in the cross-clamp patients, inotropic support was commenced in case of blood pressure problems.

In both groups of patients, arterial blood gas samples were received by the radial artery cannula before CPB, every 20 minutes during CPB, and after weaning from CPB. Blood gas analysis was performed using the Radiometer ABL 90 flex blood gas analyzer (Radiometer Medical ApS, Brønshøj, Denmark) in the operating room.

### Statistical Analysis

For data evaluation, descriptive statistics were reported in continuous variables as either mean ± standard deviation or median-interquartile range depending on the type of data distribution. Categorical variables were reported as numbers and respective percentages. To assess the normality of numerical variables, the Kolmogorov-Smirnov test was used. According to the groups, chi-square or Fisher's exact tests were used to compare categorical variables, while independent samples t-test or Mann-Whitney U test were used for continuous variables in case of normal and non-normal distributions, respectively. For intragroup preoperative and postoperative comparisons, paired t-test was used if the variables were normally distributed, and the Wilcoxon test was used if they were not. Pearson and Spearman's rho correlation coefficients were used to evaluate postoperative lactate levels with some numerical variables when the variable has normal and non-normal distributions, respectively. Univariate and multivariate logistic regression models were performed to assess the independent predictors of postoperative lactate level. Jamovi software version 1.0.7 and JASP Team software version 0.10.2 were used to perform the statistical analyzes. P<0.05 was considered statistically significant.

## RESULTS

### Baseline Patient Characteristics

A total of 100 patients were included in this study. There were 47 patients in the SCT group and 53 patients in the DCT group. The demographic and historical characteristics of the study groups are shown in Table 1. There was no difference between the groups in terms of age, gender, frequency of comorbidities, ejection fraction, glomerular filtration rate, and other laboratory parameters except hypertension, which was significantly higher in the DCT group.

**Table 1:** Preoperative demographic, laboratory and comorbidity features of SCT and DCT groups.

Variables	Group		*P*
	SCT (n=47)	DCT (n=53)	
Age (years)	65.2±9.3	63.2±10.2	0.304
Gender (%), male	38 (80.9%)	41 (77.4%)	0.856
PreOperative Ejection fraction, mean ± SD	51.3±7.7	53.2±6.5	0.182
BSA (median [IQR]), kg/m^2^	1.9 [1.7-2.1]	1.9 [1.7-2.0]	0.779
COPD (%)	13 (27.7)	7 ( 13.2)	0.120
Diabetes mellitus (%)	25 (53.2)	30 (56.6)	0.888
Hypertension (%)	22 (47.8)	38 (73.1)	0.019
LDL, mean ± SD, mg/dl	121.0±32.6	116.5±30.3	0.485
Carotid stenosis (%)	5 (10.6)	12 (22.6)	0.184
Peripheral arterial disease (%)	7 (14.9)	2 ( 3.8)	0.079
Chronic kidney disease (%)	4 ( 8.5)	11 (20.8)	0.152
HbA1c (median [IQR]), %	6.9 [5.8-8.8]	6.0 [5.5-7.5]	0.124
WBC (median [IQR]), 10^3^/µl	7,920.0 [6,940.0-8,955.0]	7,860.0 [7,160.0-9,620.0]	0.514
Hemoglobin (median [IQR]), g/dl	13.6 [11.4-15.2]	12.9 [11.6-14.3]	0.479
Hematocrit (median [IQR]),%	39.9 [34.1-44.4]	38.8 [34.2-42.8]	0.751
Neutrofil (median [IQR]), 10^3^/µl	5.2 [4.5-6.0]	5.0 [3.9-7.1]	0.822
Platelet, mean ± SD, 10^3^/µl	228.2±61.8	255.8 ± 102.4	0.101
BUN (median [IQR]), mg/dl	17.0 [12.9-24.1]	17.3 [15.1-20.4]	0.766
GFR, mean ± SD, ml/min	79.4±22.6	80.8±22.3	0.752
Albumin (median [IQR]), g/L	40.2 [38.-43.2]	40.1 [37.4-43.5]	0.972

BSA=body surface area; BUN=blood urea nitrogen; COPD=chronic obstructive pulmonary disease; DCT=double clamp technique; GFR=glomerular filtration rate; HbA1c=glycated hemoglobin; IQR=interquartile range; LDL=low-density lipoprotein cholesterol; SCT=single clamp technique; SD=standard deviation; WBC=white blood cells. Data were expressed as mean ± SD and independent samples t-test was used for normally distributed variables, while non-normally distributed variables were expressed as median [IQR] and Mann-Whitney U test was used for comparisons. Descriptive statistics for categorical variables were expressed as numbers (%) and Pearson chi-square or Fisher's exact test were used for comparisons. *P* values in italics were accepted as statistically significant *(P*<0.05).

### Intraoperative Characteristics

Since proximal anastomoses were performed with XCL in the SCT group, cross-clamp time was significantly longer in the SCT group compared to the DCT group (80.4±25.6 *vs*. 63.7±21.1 min, *P*<0.001). On the other hand, there was no difference between the groups with respect to CPB time (108.4±34.0 min in the SCT group, 110.4±33.7 min in the DCT group, *P*=0.768). LIMA was used more frequently in the DCT group than in the SCT group (*P*=0.044) (Table 2). SVG was used in the SCT group most commonly (*P*=0.049).

**Table 2:** Preoperative and postoperative blood lactate levels, operative characteristics, postoperative complications and mortality rates in SCT and DCT groups.

Variables	Group		*P*
	SCT (n=47)	DCT (n=53)	
LIMA graft use (%)	32 (68.1)	46 (86.8)	0.044
Cross-clamp time, mean ± SD, minutes	80.4±25.6	63.7±21.1	0.001
Cardiopulmonary bypass time, mean ± SD, minutes	108.4±34.0	110.4±33.7	0.768
Distal SVG (median [IQR]), number	3.0 [2.0-4.0]	3.0 [2.0-3.0]	0.049
Proximal SVG (median [IQR]), number	3.0 [2.0-3.0]	2.0 [2.0-3.0]	0.167
Distal anastomosis number, mean ± SD	3.6±1.2	3.2±0.8	0.072
Preoperative blood lactate, (median [IQR]), mmol/l	1.1 [0.8-1.4]	1.1 [0.8-1.3]	0.594
Post-CPB blood lactate, (median [IQR]), mmol/l	2.1 [1.6-2.7]	1.9 [1.7-2.4]	0.703
Pre-CPB glucose, mean ± SD, mg/dl	140.8±37.3	133.9±34.7	0.346
Post-CPB glucose, mean ± SD, mg/dl	180.3±51.2	175.6±45.6	0.636
Intraoperative mean blood pressure, mean ± SD, mmHg	60.4±2.5	60.7±2.3	0.607
Length of ICU stay (median [IQR]), days	3.0 [2.0-3.0]	3.0 [2.0-3.0]	0.962
Length of hospital stay (median [IQR]), days	7.0 [7.0-8.5]	8.0 [7.0-10.0]	0.112
Postoperative stroke (%)	0 (0.0)	5 (10.6)	0.020
Postoperative atrial fibrillation (%)	10 (21.3)	16 (30.2)	0.432
Surgical site infection (%)	3 (6.4)	3 (5.7)	0.999
Drainage during the first postoperative day (median [IQR]), ml	300.0 [215.0-350.0]	300.0 [220.0-350.0]	0.903
Mortality (%)	2 (4.3)	4 (7.5)	0.681

CPB=cardiopulmonary bypass; DCT=double clamp technique; ICU=intensive care unit; IQR=interquartile range; LIMA=left internal mammary artery; SCT=single clamp technique; SD=standart deviation; SVG=saphenous vein graft Data were expressed as mean ± SD and independent samples t-test was used for normally distributed variables, while non-normally distributed variables were expressed as median [IQR] and Mann-Whitney U test was used for comparisons. Descriptive statistics for categorical variables were expressed as numbers (%) and Pearson chi-square or Fisher's exact test were used for comparisons. *P* values in italics were accepted as statistically significant (*P*<0.05).

### Arterial Blood Lactate Measurements

**Fig. 1:**
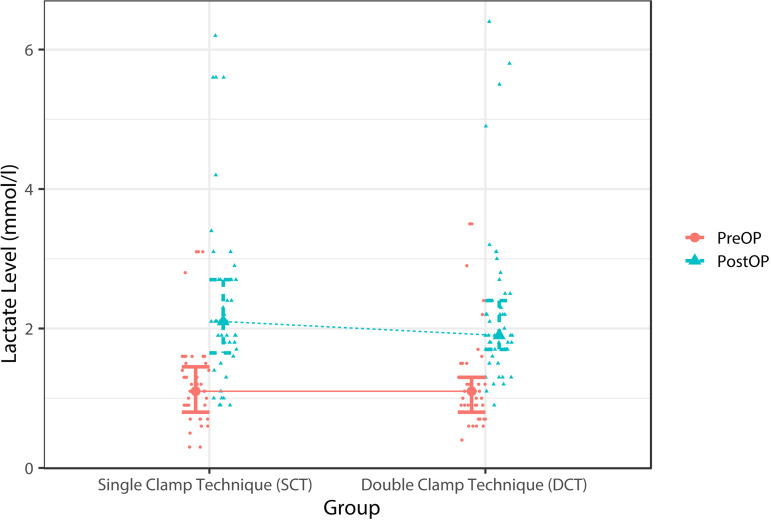
Whisker plot showing preoperative (5 minutes before cross-clamping) and postoperative (10 minutes after CPB) mean blood lactate levels in the DCT and SCT groups.

Both groups can be comparable in terms of blood lactate levels before cross-clamp (Table 2 and Figure 1). Blood lactate levels did not differ significantly between the groups evaluated ten minutes after weaning from CPB. More importantly, blood lactate evaluated ten minutes after weaning from CPB differs significantly in each group (SCT and DCT) than the cross-clamping values (*P*<0.001 for both groups). In both groups, hyperlactatemia after weaning from CPB developed in 16 patients (32%). In each group, there were 8 patients with hyperlactatemia (*P*>0.05).

### Postoperative Complications

Among the postoperative complications, the incidence of stroke was only observed in the DCT group when compared to the SCT group (*P*=0.020). Other postoperative complications were comparable between groups (Table 2). Overall, 6 patients died and there was no significant difference between groups in terms of mortality. The hyperlactatemia group (patients with blood lactate level > 3 mmol/l) had significantly more postoperative strokes compared to patients who did not have hyperlactatemia (*P*=0.028) (Table 3).

**Table 3:** Comparison of postoperative complications, intraoperative characteristics and some laboratory features among patients with postoperative serum lactate levels above and below 3 mmol/l.

	Postoperative lactate			Univariate LR		Multiple LR	
	≤3 (n=84)	>3 (n=16)	*P*	OR [95% CI]	*P*	OR [95% CI]	*P*
Postoperative stroke	2 (2.4)	3 (18.8)	0.028	-	-	-	-
Postoperative AF	23 (27.4)	3 (18.8)	0.552	-	-	-	-
Length of ICU stay	3.0 [2.0-3.0]	3.0 [2.0-3.0]	0.615	-	-	-	-
Length of hospital stay	7.0 [7.0-9.2]	8.0 [7.0-10.0]	0.230	-	-	-	-
Surgical site infection	6 (7.1)	0 (0.0)	0.586	-	-	-	-
Mortality	6 (7.1)	0 (0.0)	0.586	-	-	-	-
Drainage during the first postoperative day	300.0 [217.5-350.0]	300.0 [217.5-330.0]	0.939	-	-	-	-
Age	64.2±9.5	63.6±11.6	0.831	-	-		
HbA1c	7.0±2.0	7.7±2.5	0.311	-	-	-	-
GFR	79.0 [68.0-96.0]	88.5 [74.0-96.8]	0.483	-	-	-	-
Preoperative EF	55.0 [50.0-56.2]	52.5 [45.0-55.0]	0.289	-	-	-	-
XCL time	68.1±20.9	89.9±34.3	0.025	1.04 [1.01-1.06]	0.003	1 [0.97-1.04]	0.978
CPB time	103.6±29.3	140.6±38.7	0.002	1.04 [1.02-1.06]	< 0.001	1.04 [1.01-1.07]	0.011
LIMA graft use (%)	65 (77.4)	13 (81.2)	0.999				
Proximal SVG number (median [IQR])	2.0 [2.0-3.0]	3.0 [2.0- 3.0]	0.341	-	-	-	-
Distal anastomosis number	3.3±1.0	3.7±1.1	0.251	-	-	-	-
Hemoglobin (median [IQR])	13.2 [11.5-15.2]	13.5 [12.3-14.9]	0.402	-	-	-	-
Intraoperative need for inotropic support (%)	26 (31.0)	5 (31.2)	0.999	-	-	-	-
Intraoperative mean blood pressure	60.6±2.4	60.3±2.8	0.697	-	-	-	-
Albumin (median [IQR])	40.2 [37.3-43.5]	40.0 [39.3-41.7]	0.854	-	-	-	-
Pre-CPB glucose	136.2±35.4	142.0±39.8	0.593	-	-	-	-
Pre-CPB pO_2_	72.4±6.4	74.3±7.1	0.322	-	-	-	-

AF=atrial fibrillation; CI=confidence interval; CPB=cardiopulmonary bypass; EF=ejection fraction; GFR=glomerular filtration rate; HbA1c=glycated hemoglobin; ICU=intensive care unit; IQR=interquartile range; LIMA=left internal mammary artery; LR=logistic regression; OR=odds ratio; XCL=cross-clamp Independent samples t-test was used for normally distributed variables, while non-normally distributed variables were expressed as median [IQR] and Mann-Whitney U test was used for comparisons. Descriptive statistics for categorical variables were expressed as numbers (%) and Pearson chi-square or Fisher's exact test were used for comparisons. *P* values in italics were accepted as statistically significant (*P*<0.05).

### Correlations and Independent Associates of Blood Lactate

In the whole group, CPB time, cross-clamp time and the numbers of proximal saphenous grafts and distal anastomoses showed a significant positive correlation with the lactate level after CPB (Table 4). In multivariable regression analysis, only CPB and cross-clamp times emerged as independent predictors of hyperlactatemia after CPB (Table 3).

**Table 4:** Correlation of several operative and patient characteristics with the postoperative lactate level.

	r	*P*-value
Age	-0.018	0.859**
XCL time	0.384	<0.001**
CPB time	0.481	<0.001**
Hemoglobin	-0.010	0.923*
GFR	-0.046	0.652**
Distal anastomosis number	0.367	<0.001*
Pre-CPB glucose	0.104	0.301**
Intraoperative mean blood pressure	-0.111	0.272**
HbA1c	-0.039	0.699*
Proximal SVG number	0.310	0.002*
Pre-CPB pO_2_	0.065	0.519*

CPB=cardiopulmonary bypass; GFR=glomerular filtration rate; HbA1c=glycated hemoglobin; SVG=saphenous vein graft; XCL=cross-clamp

* Spearman's rho correlation coefficent was used.

** Pearson correlation coefficient was used.

P values in italics were considered significant.

## DISCUSSION

Prolonging CPB and cross-clamp times may have more adverse effects systemically both in the myocardium and in all other organs. In this study, we aimed to investigate the CPB and cross-clamp times of different clamping techniques and how these techniques affect the blood lactate level. In the literature, SCT and DCT do not demonstrate a superior effect on the myocardium and brain system^[5]^. Considering this, in CABG surgeries performed with CPB, we aimed to investigate the effect of applying proximal anastomoses with two different clamp techniques on the blood lactate level after CPB.

Our study showed no difference in terms of the incidence of hyperlactatemia between the SCT and DCT groups. The DCT and SCT groups were also comparable regarding to the mean blood lactate levels after CPB. CPB time emerged as the only independent associate of postoperative hyperlactatemia.

Hyperlactatemia is a common occurrence in patients undergoing various types of heart surgery, including CPB, heart transplantation, and valve surgery^[3,4,9]^. The frequency of hyperlactatemia was similar in both groups; thus, the clamping technique did not seem to confer a risk for the development of hyperlactatemia *per se.* In the whole group, the rate of hyperlactatemia after CPB was 32%. This incidence varied in previous studies between as low as 5.7%^[3]^ and as high as 71%^[4]^. We believe that this great variability results from heterogeneous patient cohorts, cut-off values for the definition of hyperlactatemia, and CPB times.

Several independent risk factors for hyperlactatemia have been proposed based on studies conducted on open-heart surgery^[1]^. The most consistent risk factors among several studies were CPB time, need for inotropic support and need for blood transfusion^[3,4,8,10,11]^. Our results showed that only CPB time was independently associated with hyperlactatemia. Besides, some of these studies also found acute kidney injury, preoperative ejection fraction, and increased glucose levels as independent determinants of postoperative hyperlactatemia. None of the latter factors remained significant in our multivariate logistic regression analysis.

Postoperative stroke occurs in about 1.3% of CABG patients^[12]^. Ischemic stroke is generally three times more common than hemorrhagic stroke^[13]^. Emboli may originate from the aorta, heart, or CPB. During the operation, the aorta is manipulated during cannulation, cross-clamping, and creation of a proximal anastomosis. Since aortic atherosclerosis is seen in more than half of all CABG patients, CABG surgery poses a considerable risk of stroke in these patients^[14,15]^. Several studies have shown that aortic SCT is associated with a lower incidence of postoperative stroke compared to DCT^[16-18]^.

During DCT, the aorta is extra manipulated during side-biting clamp application, which may dislodge more embolic material from atheromatous plaques. Our study is also in agreement with the literature, since our stroke rate was observed only in the DCT group. This situation was attributed to clamping the aorta twice. Actually, all five events of postoperative stroke occurred in DCT patients. This might arise from differences in the atherosclerotic burden of individual CABG patients. We did not screen patients to detect aortic atherosclerotic load before surgery. In fact, it is increasingly recommended to perform an epiaortic scanning before surgery^[19]^. It should also be emphasized that some studies did not found a difference in stroke incidence between SCT and DCT^[20,21]^. A recent study evaluating records of more than 50 thousand CABG surgeries concluded that the aortic clamping strategy does not affect the incidence of postoperative stroke^[22]^. Other authors also suggested that the best way to prevent postoperative stoke is not to touch the aorta at all^[23,24]^. In our study, other postoperative complications, including mortality and AF rate, were not different between groups.

We had hypothesized in the study design that different clamping techniques may affect blood lactate levels, since CPB and cross-clamp times may differ between groups. However, the results of the study did not confirm our predictions. Mean postoperative lactate levels, as well as the number of patients with hyperlactatemia, were comparable in both groups. We think that this lack of difference might be due to similar CPB times in the SCT and DCT groups. Interestingly, cross-clamp times were longer in SCT patients than in DCT patients. Still, there was no difference in lactate levels between groups. To the best of our knowledge, this is the first study in the literature attempting to unravel the impact of clamping techniques on postoperative lactate levels. Certainly, more studies are needed to draw more concrete conclusions on the subject.

Several limitations of this study are worth mentioning. First, our sample size was relatively small compared to more extensive studies in the literature. Second, we measured blood lactate levels just at the beginning of surgery and just after CPB. As carried out in some studies^[3,25]^, studying blood lactate levels at several points during the surgery and ICU stay would better reflect the trend of lactate levels. Third, we had no data on intraoperative blood transfusion practices, as well as the minimum level of arterial oxygen, which was shown to be related to the development of hyperlactatemia. Fourth, since this is a retrospective study, it was recorded that LAD distal anastomosis was performed with SVG in diseases associated with LIMA (atherosclerosis, narrower LIMA diameter, insufficent blood flow), when the records were ramdomly reassessed, in the SCT group, LIMA-LAD anastomosis was found to be lower. Due to these observations, we could not use LIMA as in the case of DCT groups. Despite these shortcomings, this study is the first in the literature to investigate the effects of different clamping techniques on hyperlactatemia after CPB.

In conclusion, despite their longer mean cross-clamp time, there was no difference in CBP time and blood lactate levels after CPB between the SCT and DCT groups. Moreover, postoperative stroke was observed only in DCT groups. Moreover, only CPB time was independently associated with the development of hyperlactatemia. Our study showed that both clamping techniques can be safely used in CABG surgery. Although cross-clamp times were significantly different for both groups, CPB times were similar. In our view, similarity of lactate levels after CPB might be dependent on similarity of CPB time. Further studies are needed to shed more light on the effect of the clamping technique on blood lactate levels.

**Table:** 

Authors' roles & responsibilities	
RÖ	Substantial contributions to the conception or design of the work; or the acquisition, analysis, or interpretation of data for the work; drafting the work or revising it critically for important intellectual content; agreement to be accountable for all aspects of the work in ensuring that questions related to the accuracy or integrity of any part of the work are appropriately investigated and resolved; final approval of the version to be published
MB	Substantial contributions to the conception or design of the work; or the acquisition, analysis, or interpretation of data for the work; drafting the work or revising it critically for important intellectual content; agreement to be accountable for all aspects of the work in ensuring that questions related to the accuracy or integrity of any part of the work are appropriately investigated and resolved; final approval of the version to be published
AİT	Drafting the work or revising it critically for important intellectual content; agreement to be accountable for all aspects of the work in ensuring that questions related to the accuracy or integrity of any part of the work are appropriately investigated and resolved; final approval of the version to be published
TE	Agreement to be accountable for all aspects of the work in ensuring that questions related to the accuracy or integrity of any part of the work are appropriately investigated and resolved; final approval of the version to be published
AT	Agreement to be accountable for all aspects of the work in ensuring that questions related to the accuracy or integrity of any part of the work are appropriately investigated and resolved; final approval of the version to be published
